# Chemical Characterization and Antibiofilm Activities of Bulbs and Leaves of Two Aglione (*Allium ampeloprasum* var. *holmense* Asch. et Graebn.) Landraces Grown in Southern Italy

**DOI:** 10.3390/molecules25235486

**Published:** 2020-11-24

**Authors:** Lucia Caputo, Giuseppe Amato, Florinda Fratianni, Raffaele Coppola, Vincenzo Candido, Vincenzo De Feo, Filomena Nazzaro

**Affiliations:** 1 Department of Pharmacy, University of Salerno, via Giovanni Paolo II, 84084 Salerno, Italy; lcaputo@unisa.it (L.C.); g.amato29@studenti.unisa.it (G.A.); 2 Institute of Food Sciences, CNR-ISA, Via Roma 64, 83100 Avellino, Italy; fratianni@isa.cnr.it; 3 Department of Agricultural, Environmental and Food Sciences, University of Molise, Via de Sanctis snc, 86100 Campobasso, Italy; coppola@unimol.it; 4 School of Agricultural, Forest, Food and Environmental Sciences, University of Basilicata, Viale dell’Ateneo Lucano, 85100 Potenza, Italy; vincenzo.candido@unibas.it

**Keywords:** *Allium ampeloprasum* var. *holmense*, aglione, polyphenols, antioxidant activity, biofilm

## Abstract

The present study was carried out to determine some biochemical characteristics, in particular the total polyphenol content and the free radical scavenging activity, of the extracts recovered from bulbs and aerial parts (these last often considered as by-products) of two landraces of *A. ampeloprasum* var. *holmense* cultivated in Southern Italy. For the first time, the capacity of the extracts of these landraces to inhibit the formation of biofilm of different Gram-negative and Gram-positive bacteria and to affect the metabolism of the cells present within the bacterial biofilm was evaluated. All extracts exhibited an amount of total polyphenols not lower than 2.86 mg/g of dried product and revealed a noteworthy antioxidant activity, with EC_50_ values not exceeding 4.95 mg. In both cases, the aerial parts extracts were more effective than the bulb extracts, which also showed a minor amount of total polyphenols. The extracts inhibited mainly the adhesive capability of *Pseudomonas aeruginosa* and *Staphylococcus aureus*, by 95.78% and 85.01%, respectively. The extracts demonstrated to inhibit also the metabolism of the bacterial cells reaching levels up to 90%. Finally, as assessed by the assays performed on the 24-h preformed biofilms, all the extracts were also capable to cause a reduction in bacterial biomass and to affect their metabolism.

## 1. Introduction

*Allium ampeloprasum* L. is a species composed of different cytotypes, which are distributed in the countries surrounding the Mediterranean Sea and that expands from North Africa, South-West Asia, to the south of England. This species consists of four gene-pools, named wild leek, European leek cultivars, Egyptian kurrat, and great headed garlic. It is a species closely related to leek (*Allium porrum* L.) and is customarily supposed as its wild progenitor [1]. A. *ampeloprasum* contains a broad series of compounds, among which polyphenols, with antioxidant properties [2,3] and recently an extract obtained from the leaves was used to synthetize silver nanoparticles used in catalytic reduction of 4-nitrophenol with antioxidant activity [4]. *Allium ampeloprasum* var. *holmense* Asch. et Graebn., also called “aglione” in Italy, has large bulblets and a much larger bulb than common garlic; in fact, it is considered more closely related to the leek than to ordinary garlic. This vegetable is a cultigen, propagated in vegetative manner all over the world, and usually shows sterile large floral umbel. The aroma of its cloves is more delicate, but very similar to that of garlic, for which it is used as substitute. In fact, it is often described as “garlic for people who don’t like garlic” [5,6,7]. Few studies reported the phytochemical composition and the biological properties of the bulbs and leaves of the “Aglione”. Recently, Ceccanti et al. [8] studied the phytochemical profile of the bulbs of the landrace of the “aglione” cultivated in Tuscany (Italy), discovering that it is capable to maintains a distinct phytochemical signature during in vitro gastrointestinal digestion, best than *A. sativum*. However, few literatures reported the antimicrobial effect of other varieties of *A. ampeloprasum*, whose effects were ascertained as due mainly to their content in diallyl thiosulphinate, methyl sulphinate, allylmethylsulphinate [9], or saponins [10]. To our knowledge, there is a complete lack of information about the capacity of the extracts of *A. ampeloprasum* var. *holmense* to inhibit the growth of bacteria and to avoid or limit the adhesion capacity of bacteria and the following biofilm formation by bacteria and affect the metabolism of the cells present into the bacterial biofilm, which versatile scenario takes also place to the differences occurring in the susceptibility of planktonic and biofilm bacterial cells to antimicrobial agents. Pellegrini and Ponce [11] studied the antibacterial activity of the aerial parts extracts of *Allium porrum*, mainly related to their quorum quenching capacity and biofilm inhibitory action, and found that these extracts were capable to inhibit the bacterial biofilm of *Escherichia coli* ATCC 25158 at a concentration of 268.75 mg/mL. Therefore, the present study was carried out to determine the total polyphenol content of the extracts recovered by bulbs and aerial parts (these last often considered as by-products) of two landraces of *A. ampeloprasum* var. *holmense* grown in Southern Italy and its antioxidant and anti-biofilm activities. The antioxidant activity was evaluated using the DPPH assay and the inhibitory activity of the extracts on the bacterial adhesive capability and biofilm activity was evaluated against two Gram-positive (*Staphylococcus aureus* and *Listeria monocytogenes*) and three Gram-negative (*Escherichia coli*, *Pseudomonas aeruginosa* and *Pectobacterium carotovorum*) bacterial strains. The use of bioactive extracts, rich in polyphenols and with antioxidant activity, recovered from unused portions of vegetal byproducts, if possessing also anti-pathogenic activity against a wide range of bacteria -including those more difficult to treat- could be proposed as an alternative not only to the use of the conventional drugs but also in the design of novel preserving agents, capable to limit the deterioration of foods during their shelf-life, maintaining or even prolonging their quality and safety, with benefits for the consumer health.

## 2. Results and Discussion

### 2.1. Total Polyphenols and Free Radical Scavenging Capacity

The yield, total polyphenol content and the free radical scavenging capacity of the extracts of bulbs and aerial parts of the landraces ‘Irsina’ and ‘Contursi Terme’ (’Contursi T.’) of *A. ampeloprasum* var. *holmense* are shown in Table 1.

The content of total polyphenols resulted higher in the aerial parts than in the bulbs. The aerial parts of the ‘Contursi T.’ landrace presented a content of total polyphenols almost double respect to that found in the bulbs (5.364 mg/g of air-dried product and 2.867 mg/g of air-dried product, respectively). Bulbs of the ‘Contursi T.’ landrace exhibited also the lowest yield, justifying, perhaps, the lowest content of total polyphenols (TPs), indeed. The gap between the content of TPs in the aerial parts and bulbs of ‘Irsina’ not was so marked, although TPs content of the aerial parts resulted higher than that of the bulbs (5.266 mg/g of air-dried product and 4.438 mg/g of air-dried product, respectively). The presence of a high amount of total polyphenols in the leaves of *Allium* species has been reported also by Pellegrini and Ponce [11], who found 262.66 mg of total polyphenols in 100 g of the leaves of dried leek. The content we found proved inferior to that reported by Lu and co-workers [3] for bulbs and cloves of *A. ampeloprasum*. However, as is well known, the amount of secondary metabolites is affected by several factors, including the variety, the method of extraction, the harvesting time [11]. High levels of polyphenols have been reported also by Ceccanti and co-workers [8] for bulbs of of *A. ampeloprasum* var. *holmense* cultivated in Tuscany (Italy).

The capacity of the extracts to act as free radical scavenger was also evaluated. Results (Table 1) indicated that all the extracts were effective to act as antioxidants. The extracts of aerial parts of both landraces exhibited a greater antioxidant power, that, in the case of ‘Contursi T.’ reached a value of EC_50_ = 0.872 mg. In the case of ‘Irsina’, EC_50_ did not exceed 1.033 mg. Such correspondence was also evident for the extracts of the bulbs that showed a weaker scavenging capacity, with EC_50_ values never superior to 4.6 mg. A strong antioxidant activity was reported for several *Allium* species. It was shown to be very high in more than 40 cultivars of *A. sativum* cultivated in China [12] and in populations of *A. ampeloprasum* from Spain [1]. In some cases, the antioxidant activity of *A. ampeloprasum* resulted higher when compared to that exhibited by some cultivars of Italian *A. sativum* [13]. The biological and health-promoting properties of garlic is due primarily to its polyphenols and organosulfur compounds [14]. However, these latter compounds are considered extremely unstable and susceptible to further transformation into volatile compounds. Attention has thus been paid to polyphenols of garlic due to their potential role in health-related benefits to humans, not only as antioxidants, but also as prebiotics [15] and as natural agents fighting the growth of pathogenic bacteria [16].

### 2.2. Antibacterial Activity

The minimal inhibitory concentration (MIC), necessary to block the growth of the bacteria of the extracts, was evaluated through the resazurin test. Results are reported in Table 2.

By the whole, the extracts exhibited an inhibitory activity. *L. monocytogenes* showed more resistance: in fact, the MIC value was higher than 20 mg/mL for both the ‘Irsina’ extracts, whereas the extracts of the bulbs and aerial parts of ‘Contursi T.’ were capable to majorly act on this bacterial strain (MIC = 10 mg/mL and 6 mg/mL, respectively). Probably the bulbs, but mainly the aerial parts of this landrace contained a metabolic profile that might affect in particular way the growth of *L. monocytogenes*. The extracts from the bulbs of both landraces were more effective against *E. coli* than those of the aerial parts. The activity exhibited by the extracts against *S. aureus* and *P. aeruginosa* (that was also the most sensitive strain) and *P. carotovorum* was similar.

Our results indicated the antibacterial activity of *A. ampeloprasum* var. *holmense*, and confirmed the potentialities of *Allium* species to be used to fight both against Gram-positive and Gram-negative bacteria [17,18,19,20]. However, scarce consideration has been given to their antibiofilm potential [21,22]. In our experiments, the extracts were tested to evaluate their strength in contrasting both the adhesive capacity to a biomaterial surface, a step during which bacteria are still susceptible to antimicrobial agents (Table 3) and the metabolism of the bacterial cells during such step (Table 4). *S. aureus* and *P. aeruginosa* were the most sensitive bacteria (Table 3). The activity was also more powerful compared to that exhibited by the ethanolic extracts of aerial parts and bulbs of *A. orientale,* which were effective in contrasting the formation of the biofilm of these two bacteria but at higher concentration [23]. The extract of the ’Irsina’ bulb determined, already at a concentration of 0.4 mg/mL, an inhibition of 43.7% and an almost total inhibition (95.78%) at a concentration of 2 mg/mL. A similar efficiency (89.17%) was observed with 2 mg/mL for ‘Irsina’ extract of aerial parts vs. *P. aeruginosa*. The inhibitory activity vs. this strain was also remarkably exhibited by both extracts of the ‘Contursi T.’ landrace, although, in this case, the bulb extract was less effective than the bulb extract of ‘Irsina’. The aerial parts extract of the ‘Contursi T.’ landrace was more effective than that obtained from the ‘Irsina’ landrace when used at the lower concentration (0.4 mg/mL), causing an inhibition of 53.93%, a value more than double the inhibition determined by the extract from ‘Irsina’ aerial parts.

*S. aureus* also proved to be very sensitive to the action of the extracts, which caused level of inhibition by 73.25% (‘Irsina’ aerial parts extract) and 85.01% (‘Contursi T.’ bulb extract, at 2 mg/mL). In this case, contrary to what observed in the test conducted vs. *P. aeruginosa*, the bulb exract of ‘Contursi T.’ proved to be more effective than the extract of the ‘Irsina’ bulb, with a Δ in the inhibitory action of 20.25%. The inhibitory efficacy exhibited by the extracts vs. *E. coli* was much less incisive, and a maximum of inhibition at 31.44% was obtained, at the higher concentration used of the aerial part extract of ‘Contursi T.’ This inhibition was reported also for *A. orientale* [23] and *A*. *sativum* [24], although with different effectiveness. Therefore, ethanolic and methanolic extracts of *A. sativum* exhibited capacity to block the biofilm of bacteria, including *S. aureus*, *P. aeruginosa,* and *E. coli,* suggesting that these extracts might be applied as antimicrobial agents against these pathogens, particularly in biofilm forms [25]. This could be of particular aid in the preliminary treatment of the surface of test tubes and catheters, generally attacked by different pathogenic bacteria as demonstrated by the *A. sativum* essential oil [26]. *P. carotovorum* was sensitive to the action of the ‘Irsina’ extracts, resulting the aerial parts more effective than bulbs (52.24% and 37.47%, respectively). The extracts of the landrace ‘Contursi T.’ exhibited an opposite behavior, resulting bulbs much more powerful than aerial parts in inhibiting the formation of the biofilm (73.42% and 6.77%, respectively). *L. monocytogenes* proved to be the most resistant strain, being sensitive only to the action of the ‘Contursi T.’ bulb extract, which determined an inhibition of 43.12%. The influence exerted by the extracts on the metabolism of the bacterial cells seemed in some cases to trace the behavior exhibited by the extracts on the ability to inhibit the adhesive capacity of the bacteria (Table 4).

This was evident in the case of *P. aeruginosa*, against which the extracts of ‘Irsina’, which had proved effective in inhibiting the adhesive capability of this bacterium, were even more effective in limiting the metabolic changes occurring in the bacterial cells, so much so that 0.4 mg/mL of the ‘Irsina’ bulb extract determined 43.7% inhibition of the biofilm but its ability to inhibit bacterial cell metabolism within the biofilm increased up to 91.78%. A similar action was exercised by the extract of ‘Irsina’ aerial parts that was able not only to limit the adhesion of bacteria but also to inhibit the metabolic cell changes with three times higher vigor. The extracts of ‘Contursi T.’ exhibited, vs. *P. aeruginosa*, a similar metabolic inhibition. *S. aureus*, which had also been shown to be sensitive to the biofilm-inhibitory activity of all extracts, was much more sensitive to their metabolic-inhibitory action, with an observed lack of metabolic change that determines the transformation of bacterial cells and concurs to a greater pathogenicity of bacteria, which become more resistant to conventional antibiotics. The other three bacteria tested also proved more sensitive to the action of extracts on cell metabolism, as evidenced by the data obtained from the MTT test. In fact, *L. monocytogenes*, which also resisted to the action of the extracts in its adhesive capacity, was instead much more sensitive to the activity of the extracts on its metabolism. The only extract that somehow counteracted the adhesion of *L. monocytogenes* (the ‘Contursi T.’ bulb extract), showed a minor impact on the metabolic changes occurring during this phase.

When the tests were carried on preformed 24-h biofilms, the effects were variable. Results are shown in Table 5 and Table 6. As assessed by the CV assay, performed on the 24-h preformed biofilms, all the extracts were able to cause a reduction in bacterial biomass (Table 5).

Some extracts, such as that from the ‘Contursi T.’ aerial parts, were able to determine a reduction of 79.73% vs. *S. aureus*, already at a concentration of 0.4 mg/mL. This data is particularly interesting, considering how *S. aureus* today represents a microorganism particularly difficult to eradicate and is one of the most problematic bacteria from a medical-clinical point of view. When the extracts were used at the highest concentration, the inhibitory action on the biomass was superior to 80%. The effect was less pronounced only against *P. aeruginosa*, with percentages ranging from 33.61% (in the presence of 2 mg/mL of ‘Contursi T.’ aerial parts extracts) to 78.62% (when the test was carried with 2 mg/mL of the ‘Irsina’ bulb extracts). Although the CV assay is used as an indicator of the attached biomass in a biofilm, it cannot reveal the metabolic status of the cells. Thus, we performed also onto the 24-h preformed biofilm the MTT test, using the tetrazolium salt that, in the presence of metabolically active cells, is generally reduced into a product that can be measured colourimetrically. Results of the MTT assay (Table 6) indicated that the extracts were able to inhibit the metabolism of the cells present within the preformed biofilms but that a concentration higher than 0.4 mg mL was almost always required.

In any case, the extracts were able to act even on 24-h preformed biofilms although concentrations equal to 2 mg/mL seemed necessary to have the double effect, i.e., the reduction of biomass and the prevention of the metabolic activity of the cells.

There are different stages in the process of biofilm formation, in the first of which, planktonic cells create reversible attachment to a biomaterial surface and/or host cell surface and are still susceptible to antimicrobial agents. Then, the bacterial adhesion to surface becomes irreversible and the cells begin to secrete exopolysaccharides that decrease the amount of antimicrobial available to interact with biofilm [27]. Our results ascertained the potential use of the extracts of *A. ampeloprasum* that in our experiments resulted also much more effective than those obtained from *A. porrum* [11] or *A. sativum* [28] in inhibiting the formation of biofilm, although the times considered for the biofilm formation were different. Furthermore, they confirmed once again the enormous role that the vegetal extracts can exhibit in fighting not only to act ab origine on the adhesion of cells, preclude of biofilm formation, but also in exerting some mechanisms capable to affect the surface of bacterial cells or inside them, once the biofilm has formed (in our case, considering 24-h of preformed biofilms) and the microbial cells are trapped in [25,28,29]. Our data are useful for both valorizations of such landraces and for their possible utilization in larger food preparations, also outside the original area. Furthermore, having a huge amount of waste plant material available must no longer be seen as a problem but as a great opportunity. The extraction of bioactive substances of vegetables can be valued as pharmaceuticals and other products, such as functional ingredients for food, health care, and cosmetics purposes.

## 3. Materials and Methods

### 3.1. Plant Material

Ten whole plants of ‘Irsina’ and ‘Contursi T.’ landraces were collected in May 2020 in an experimental field in Pontecagnano Faiano (Salerno Province, Southern Italy). Plants of both landraces were grown on a fine-texture soil, previously ploughed and fertilized. Planting took place in the middle of November 2019 by using 100 uniform size cloves, spaced 20 cm on rows 50 cm apart (10 plants m^−2^). Voucher specimens were deposited in the Herbarium of Medical Botany Laboratory, at the University of Salerno.

### 3.2. Extraction Procedure

Bulbs and aerial parts of the two landraces were separated, cleaned, and air-dried. Each plant part was extracted at room temperature with methanol (150 g/L) (Sigma, Milan, Italy). Extractions were carried out for 5 days and 3 times, with frequent agitation. The supernatant was then filtered and dried under reduced pressure in a rotary system (Rotavapor Heidolph, Schwabach, Germany) to obtain dried extracts. The yields are reported in Table 1.

### 3.3. Total Polyphenols Determination

The content of total polyphenols was evaluated at λ = 760 nm (Cary 50 Uv/Vis spectrophotometer, Varian-Agilent, Cernusco sul Naviglio, Italy), with Folin-Ciocalteau reagent. Gallic acid was used as standard [30], based also on the work of Nazzaro and co-workers [16]. The results were expressed as mg gallic acid equivalent (GAE)/g of air-dried sample.

### 3.4. Free Radical Scavenging Capacity

The free radical scavenging activity was determined following the method of Brand-Williams et al., using the stable radical 2,2-diphenyl-1-picrylhydrazyl (DPPH) assay [31]. The analysis was performed in microplates by adding 7.5 µL of an extract to 303 µL of a methanolic DPPH solution (153 mM). Next, the absorbance at 517 nm was measured (Cary 50 MPR, Varian, Cernusco sul Naviglio, Italy). The absorbance of DPPH in the absence of antioxidants (control sample) was used to determine the baseline value. The extract concentration providing 50% of the radical scavenging activity was assumed as EC_50_.

### 3.5. Antibacterial Activity

#### 3.5.1. Microorganisms and Culture Conditions

*Listeria monocytogenes* ATCC 7644, EHEC, *Escherichia coli* DSM 8579, *Pseudomonas aeruginosa* DSM 50071, *Pectobacterium carovotorum* DSM 102074 and *Staphylococcus aureus* subsp. *aureus* ATCC 25923 were used as test bacterial strains. Bacteria were cultured in Luria–Bertani (LB) broth (Sigma, Milan, Italy) for 18 h at 37 °C and 80 rpm (Corning LSE, Pisa, Italy). *P. carotovorum* was grown at 28 °C and 80 rmp for 18 h (Corning LSE, Pisa, Italy).

#### 3.5.2. Minimal Inhibitory Concentration (MIC)

The MIC values were calculated through the application of the resazurin microtiter-plate assay [32]. Samples were dissolved in sterile Dimethyl sulfoxide (DMSO). Two-fold serial dilutions were prepared to have 50 μL of the samples in serially descending concentrations in each well. Thirty-five μL of 3.3 × strength iso-sensitized broth and 5 μL of resazurin, as indicator solution, were added to have a final volume/well of 240 μL with several volumes of sterile Luria Bertani broth. Finally, 10 μL of bacterial suspension was added to each well to reach a concentration of about 5 × 10^5^ cfu/mL. Sterile DMSO and ciprofloxacin (Sigma–Aldrich, Milan, Italy, 1 mg/mL in DMSO) were the negative and positive controls, respectively. Microtiter-plates were prepared in triplicate and incubated at 37 °C for 24 h. Microtiter-plates containing *P. carotovorum* were incubated at 28 °C. The lowest concentration causing a color change (from dark purple to colorless) showed the MIC value.

#### 3.5.3. Bacterial Adhesion Ability

The effect of the extracts to inhibit the bacterial adhesion ability was evaluated in flat-bottomed 96-well microtiter plates following the methods of O’ Toole and Kolter [33] and Caputo et al. [34], using doses ranging from 0.4 to 2 mg/mL. In each well, the overnight bacterial cultures were adjusted to 0.5 McFarland (1.5 × 10^7^ cells/mL, Densitometer cell density turbidity 0.3–15.0 McFarland, CAMLAB, Cambridge, United Kingdom) with fresh culture broth. Then, 10 μL of the diluted cultures were distributed in each well, and different volumes of the extracts and Luria-Bertani broth were added, to reach a final volume of 250 μL/well. Microplates were completely covered with parafilm tape, to prevent the evaporation of samples and incubated for 48 h at different temperatures (depending on the strain). Planktonic cells were removed, and the attached cells were gently washed twice with sterile physiological saline solution. Then, 200 μL of methanol was added to each well and retained for 15 min to fix the sessile cells. Methanol was then discarded, and each microtiter plate was left until complete dryness of samples. The staining of the adhered cells was performed with 200 μL of 2% *w/v* crystal violet solution that was added to each well, left for 20 min and discarded. Wells were gently washed with sterile physiological solution and left to dry. Two hundred microliters of glacial acetic acid 20% *w/v* were added to allow the release of the bound dye. The absorbance was measured at OD = 540 nm (Varian Cary). The percent value of biofilm inhibition was calculated with respect to control (cells grown without the presence of the samples). Triplicate tests were done, and the average results were taken for reproducibility.

#### 3.5.4. Metabolic Activity of Biofilm Cells

The effects of different doses of the extracts (0.4–2.0 mg/mL) on the metabolic activity of biofilm cells was evaluated through the 3-(4,5-dimethylthiazol-2-yl)-2,5-diphenyltetrazolium bromide (MTT) colorimetric method according to Kairo et al. [35] and Fratianni et al. [36], using 96-well microtiter plates. The overnight bacterial cultures were adjusted to 0.5 McFarland and treated as described in Section “Biofilm Inhibitory Activity”. After 48 h incubation, bacterial suspension was removed and 150 μL of PBS and 30 μL of 0.3% of MTT (Sigma, Milan, Italy) were added, keeping microplates at 37 °C. After 2 h, the MTT solution was removed and two washing steps were performed with 200 μL of sterile physiological solution. Then, 200 μL of DMSO were added to let the dissolution of the formazan crystals that were measured at OD = 570 nm after 2 h (Varian Cary Spectrophotometer model 50 MPR, Cernusco sul Naviglio, Italy). Triplicate tests were carried out and the average results were taken for reproducibility.

#### 3.5.5. Effect of the Extracts on Preformed Biofilms

The effect of different doses (0.4–2 mg/mL) of the extracts on the bacterial biofilm was evaluated in flat-bottomed 96-well microtiter plates modifying the protocol of Kang et al. [37]. In each well (final volume: 0.25 mL), 10 μL of the overnight bacterial cultures were adjusted to 0.5 McFarland with Luria-Bertani (LB) culture broth. Plates were incubated at 37 °C and 28 °C for 24 h, depending on the strain. Then, the broth was accurately removed, to eliminate the non-adhered cells and the plates were washed twice with sterile physiological solution. Next, different volumes of fresh LB broth and of the extracts were added, to reach a final volume of 250 μL/well. Microplates were completely covered with parafilm tape, to prevent the evaporation of samples and incubated for 24 h at different temperatures (depending on the strain). The broth was once again discarded, and the attached cells were gently washed twice with sterile physiological saline solution. Then, the protocol already indicated in Section 3.5.3 was used. The absorbance was measured at OD = 540 nm (Varian Cary). The percent value of biofilm inhibition was calculated with respect to control (cells grown without the presence of the samples). Triplicate tests were done, and the average results were taken for reproducibility.

#### 3.5.6. Effect of the Extracts on the Cell Metabolic Activity in Preformed Biofilms

The effect of different doses (0.4–2 mg/mL) of the extracts on the bacterial metabolism within a 24-preformed biofilm was evaluated in flat-bottomed 96-well microtiter plates. In each well (final volume: 0.25 mL), 10 μL of the overnight bacterial cultures were adjusted to 0.5 McFarland (1.5 × 10^7^ cells/mL) with Luria-Bertani (LB) culture broth. Plates were incubated at 37 °C and 28 °C for 24 h, depending on the strain. Then, the broth was accurately removed, to eliminate the non-adhered cells and the plates were washed twice with sterile physiological solution. Next, different volumes of fresh LB broth and of the extracts were added, to reach a final volume of 250 μL/well. Microplates were completely covered with parafilm tape, to prevent the evaporation of samples and incubated for 24 h. Cultured broth was once again discarded, and the attached cells were gently washed twice with sterile physiological saline solution. Then, the protocol already described in the Section 3.5.4 was used. The dissolution of the formazan crystals was measured also in this case at OD = 570 nm after 2 h (Varian Cary). Triplicate tests were carried out and the average results were taken for reproducibility.

## 4. Conclusions

This research provides some insight into two landraces of *Allium ampeloprasum* var. *holmense*, once used in Southern Italian traditional foods. The data recorded in this research once again confirm the importance of vegetal biodiversity and the study of local plant accessions for the research of possible functional foods or basis from which to extract ingredients with important biological activities, in this case the antibacterial one. From the results, we could suggest a higher diffusion of the cultivation of these so-called neglected species, and also for the utilization of their aerial parts, often discarded, which, due for their content of polyphenols and their antioxidant activity, could be suggested as a component of the diet. Although these are local species, the work performed stimulates the study of the biodiversity, for the search of novel substances useful as antibacterial and anti-biofilm activity, to be considered not only, e.g., in natural medicine or as an effective substitute for conventional antibacterial drugs, but also in food preservation, where *A*. *sativum* or *A. cepa* are used. Our results suggest promoting the cultivation of the “aglione” in Southern Italy where there are several local populations of *A. ampeloprasum* var. *holmense,* but generally grown on small surfaces, mostly in vegetable family gardens, and there is a risk that they will be lost.

## Figures and Tables

**Table 1:** The percent yield on fresh weight basis, amount of total polyphenols (as mg GAE/g of air-dried product) and the free radical DPPH scavenging capacity exhibited by the extracts of bulbs and aerial parts of *A. ampeloprasum* var. *holmense* ‘Irsina’ and ‘Contursi T.’ landraces. The results are the mean (± standard deviation) of three independent experiments.

	Yield (%)	Total Polyphenols mg (GAE)/g ADP	Free radical Scavenging Capacity EC_50_ (mg)
‘Irsina’ bulbs	4.75	4.438 (0.243)	2.11 (0.082)
‘Irsina’ aerial parts	5.01	5.266 (0.298)	1.033 (0.031)
‘Contursi T.’ bulbs	0.78	2.867 (0.166)	4.595 (0.303)
‘Contursi T.’ aerial parts	5.20	5.364 (0.17)	0.872 (0.013)

**Table 2:** Minimal inhibitory concentration (mg/mL ± SD) exhibited by the extracts of aerial parts and bulbs of the two landraces of *A. ampeloprasum* against the pathogen strains *E. coli* (EC), *L. monocytogenes* (LM), *P. carotovorum* (PC), *P. aeruginosa* (PA) and *S. aureus* (SA). Data are the mean of three independent experiments.

	‘Irsina’ Bulbs	‘Irsina’ Aerial Parts	‘Contursi T.’ Bulbs	‘Contursi T.’ Aerial Parts
EC	6 (0.3)	18 (1.2)	8 (0.2)	15 (0.5)
LM	>20	>20	10 (0.5)	6 (0.1)
PC	6 (0.5)	5 (0.5)	18 (0.2)	4 (0.2)
PA	3 (0.05)	4 (0.1)	4 (0.2)	4 (0.2)
SA	5 (0.2)	4 (0.5)	5 (0.2)	4 (0.2)

**Table 3:** Percent inhibition of the adhesion of bacterial cells caused by the extracts of bulbs and aerial parts of ‘Irsina’ and ‘Contursi T.’ landraces. The data are expressed as percentage respect to the control (value 0). Results are the mean of three experiments (±SD). EC: *E. coli*; LM: *L. monocytogenes*; PC: *P. carotovorum*; PA: *P. aeruginosa*; SA: *S. aureus*. 0.4: 0.4 mg/mL of the extract; 1: 1.0 mg/mL of the extract; 2: 2.0 mg/mL of the extract.

	‘Irsina’ Bulbs			‘Irsina’ Aerial Parts			‘Contursi T.’ Bulbs			‘Contursi T.’ Aerial Parts		
	0.4	1	2	0.4	1	2	0.4	1	2	0.4	1	2
	10.24 (0.87)	17.44 (1.33)	18.32 (1.67)	1.75 (0.05)	5.67 (0.43)	25.35 (1.15)	5.78 (0.22)	7.74 (0.26)	13.75 (1.33)	2.45 (0.02)	23.24 (1.33)	31.44 (1.67)
LM	0 (0)	0 (0)	0 (0)	0 (0)	0 (0)	0 (0)	0 (0)	17.57 (1.33)	43.12 (2.67)	0 (0)	0 (0)	0 (0)
PC	0 (0)	0 (0)	37.47 (1.67)	0 (0)	0 (0)	52.24 (3.33)	0 (0)	30.36 (1.67)	73.42 (1.67)	0 (0)	2.33 (0)	6.77 (0.57)
PA	43.71 (1.67)	88.07 (1.67)	95.78 (1.33)	21.03 (1.03)	82.67 (1.67)	89.17 (1.33)	44.50 (1.35)	71.49 (1.21)	72.60 (1.35)	53.93 (1.67)	70.93 (1.67)	87.57 (1.67)
SA	17.16 (1.04)	58.14 (3.33)	64.76 (5.94)	15.13 (0.99)	15.76 (1.57)	73.25 (0.57)	26.25 (1.67)	38.07 (1.67)	85.01 (1.67)	20.56 (0.57)	49.74 (1.67)	68.67 (1.67)

**Table 4:** Inhibition of the metabolic activity exhibited by the bacterial cells in the presence of different concentrations of the extracts of bulbs and aerial parts of ‘Irsina’ and Contursi T.’ landraces. The data are expressed as percentage respect to the control (assumed as 0%). Results are the mean of three experiments (±SD). EC: *E. coli*; LM: *L. monocytogenes*; PC: *P. carotovorum*; PA: *P. aeruginosa*; SA: *S. aureus*. 0.4: 0.4 mg/mL of the extract; 1: 1.0 mg/mL of the extract; 2: 2.0 mg/mL of the extract.

	‘Irsina’ Bulbs			‘Irsina’ Aerial Parts			‘Contursi T.’ Bulbs			‘Contursi T.’ Aerial Parts		
	0.4	1	2	0.4	1	2	0.4	1	2	0.4	1	2
EC	25.39 (3.05)	28.95 (3.85)	39.62 (1.57)	8.44 (0.89)	12.44 (0.97)	45.91 (1.57)	11.99 (1.33)	27.71 (2.57)	39.56 (2.57)	9.76 (1.02)	42.13 (3.33)	51.87 (4.05)
LM	0 (0)	4.58 (0.33)	6.35 (0.15)	0 (0)	0 (0)	0 (0)	0 (0)	7.42 (0.57)	13.08 (0.57)	0 (0)	4.87 (0.03)	17.40 (2.33)
PC	0 (0)	1.73 (0.09)	30.69 (2.57)	0 (0)	0 (0)	17.12 (1.57)	15.97 (0)	35.47 (2.33)	46.76 (2.25)	0 (0)	31.90 (2.05)	51.09 (2.67)
PA	91.78 (1.33)	95.39 (0.57)	97.02 (1.33)	64.87 (2.33)	71.18 (1.57)	81.17 (2.57)	36.79 (2.67)	53.17 (2.57)	84.19 (2.33)	50.97 (4.03)	70.5 (2.55)	96.67 (1.33)
SA	78.74 (2.57)	88.66 (1.34)	89.75 (1.25)	85.61 (1.39)	90.5 (2.05)	91.43 (1.67)	85.73 (1.57)	89.61 (2.14)	90.45 (1.67)	87.87 (1.33)	89.81 (2.67)	92.13 (2.33)

**Table 5:** Inhibitory activity exhibited by the extracts of bulbs and aerial parts of ‘Irsina’ and ‘Contursi T.’ landraces on 24-h preformed biofilm. The data are expressed as percentage respect to the control (value 0). Results are the mean of three experiments (±SD). EC: *E. coli*; LM: *L. monocytogenes*; PC: *P. carotovorum*; PA: *P. aeruginosa*; SA: *S. aureus*. 0.4: 0.4 mg/mL of the extract; 1: 1.0 mg/mL of the extract; 2: 2.0 mg/mL of the extract.

	‘Irsina’ Bulbs			‘Irsina’ Aerial Parts			‘Contursi T.’ Bulbs			‘Contursi T.’ Aerial Parts		
	0.4	1	2	0.4	1	2	0.4	1	2	0.4	1	2
EC	50.80 (4.98)	52.71 (1.30)	53.78 (2.84)	17.13 (2.38)	55.53 (0.21)	61.41 (3.55)	4.40 (0.67)	23.54 (0.67)	41.55 (3.75)	22.17 (1.15)	28.44 (2.44)	83.65 (1.43)
LM	9.88 (4.12)	44.78 (1.75)	71.27 (1.34)	23.71 (0.16)	40.67 (0.25)	48.05 (2.52)	46.70 (1.63)	65.77 (1.01)	51.38 (2.24)	36.48 (4.06)	61.20 (1.20)	81.50 (1.47)
PC	73.49 (1.3)	79.28 (0.75)	86.90 (1.23)	8.24 (0.79)	35.91 (0.91)	85.83 (2.36)	64.04 (1.61)	67.55 (2.22)	70.37 (2.29)	66.56 (1.11)	73.74 (4.34)	83.57 (1.35)
PA	43.91 (6.26)	56.81 (3.2)	78.62 (1.17)	45.03 (0.94)	55.41 (3.56)	59.92 (5.32)	7.65 (1.67)	58.89 (4.55)	67.21 (4.75)	0 (0)	27.61 (3.38)	33.61 (1.37)
SA	28.28 (4.44)	48.77 (3.88)	83.19 (1.26)	43.11 (0.21)	57.85 (1.83)	87.52 (1.67)	38.93 (3.57)	69.53 (1.38)	80.67 (1.11)	79.73 (1.22)	75.51 (1.44)	84.54 (1.67)

**Table 6:** Inhibitory activity exhibited by the different concentrations of the extracts of bulbs and aerial parts of ‘Irsina’ and ‘Contursi’ landraces on the bacterial metabolism within the 24-h preformed bacterial biofilms. The data are expressed as percentage respect to the control (assumed as 0%). Results are the mean of three experiments (±SD). EC: *E. coli*; LM: *L. monocytogenes*; PC: *P. carotovorum*; PA: *P. aeruginosa*; SA: *S. aureus*. 0.4: 0.4 mg/mL of the extract; 1: 1.0 mg/mL of the extract; 2: 2.0 mg/mL of the extract.

	‘Irsina’ Bulbs			‘Irsina’ Aerial Parts			‘Contursi T.’ Bulbs			‘Contursi T.’ Aerial Parts		
	0.4	1	2	0.4	1	2	0.4	1	2	0.4	1	2
EC	0 (0)	0 (0)	36.98 (2.02)	15.12 (2.13)	38.38 (4.2)	61.91 (1.82)	0 (0)	31.33 (0.83)	40.66 (0.94)	15.95 (0.85)	23.10 (1.36)	38.73 (1.43)
LM	0 (0)	0 (0)	33.52 (0.69)	27.70 (1.96)	39.52 (2.71)	60.94 (2.43)	0 (0)	0 (0)	62.73 (1.38)	0 (0)	29.60 (1.12)	37.76 (1.47)
PC	0.95 (0.05)	49.46 (3.36)	75.94 (0.73)	35.29 0.77()	38.73 (0.94)	73.50 0.79()	0.7 (0.01)	11.32 (0.15)	75.91 (0.73)	31.84 (0.64)	59.83 (0.74)	70.35 (0.57)
PA	0 (0)	42.74 (1.21)	82.66 (2.47)	34.79 (1.54)	57.43 (1.60)	57.75 (1.73)	0 (0)	25.22 (0.24)	67.23 (1.32)	0 (0)	21.22 (0.73)	32.34 (0.81)
SA	19.60 (0.05)	37.91 (1.77)	64.87 (2.18)	25.51 (0.72)	47.72 (1.91)	64.44 (1.31)	0 (0)	58.80 (1.43)	72.31 (1.13)	0 (0)	32.75 (1.44)	51.10 (0.26)
